# Chloroplast Ribosomes Interact With the Insertase Alb3 in the Thylakoid Membrane

**DOI:** 10.3389/fpls.2021.781857

**Published:** 2021-12-23

**Authors:** Bernd Ackermann, Beatrix Dünschede, Björn Pietzenuk, Bo Højen Justesen, Ute Krämer, Eckhard Hofmann, Thomas Günther Pomorski, Danja Schünemann

**Affiliations:** ^1^Molecular Biology of Plant Organelles, Faculty of Biology and Biotechnology, Ruhr University Bochum, Bochum, Germany; ^2^Department of Molecular Genetics and Physiology of Plants, Faculty of Biology and Biotechnology, Ruhr University Bochum, Bochum, Germany; ^3^Department of Molecular Biochemistry, Faculty of Chemistry and Biochemistry, Ruhr University Bochum, Bochum, Germany; ^4^Protein Crystallography, Faculty of Biology and Biotechnology, Ruhr University Bochum, Bochum, Germany

**Keywords:** Oxa1/YidC/Alb3 protein family, ribosomes, thylakoid membrane biogenesis, cotranslational protein transport, nanodiscs

## Abstract

Members of the Oxa1/YidC/Alb3 protein family are involved in the insertion, folding, and assembly of membrane proteins in mitochondria, bacteria, and chloroplasts. The thylakoid membrane protein Alb3 mediates the chloroplast signal recognition particle (cpSRP)-dependent posttranslational insertion of nuclear-encoded light harvesting chlorophyll a/b-binding proteins and participates in the biogenesis of plastid-encoded subunits of the photosynthetic complexes. These subunits are cotranslationally inserted into the thylakoid membrane, yet very little is known about the molecular mechanisms underlying docking of the ribosome-nascent chain complexes to the chloroplast SecY/Alb3 insertion machinery. Here, we show that nanodisc-embedded Alb3 interacts with ribosomes, while the homolog Alb4, also located in the thylakoid membrane, shows no ribosome binding. Alb3 contacts the ribosome with its C-terminal region and at least one additional binding site within its hydrophobic core region. Within the C-terminal region, two conserved motifs (motifs III and IV) are cooperatively required to enable the ribosome contact. Furthermore, our data suggest that the negatively charged C-terminus of the ribosomal subunit uL4c is involved in Alb3 binding. Phylogenetic analyses of uL4 demonstrate that this region newly evolved in the green lineage during the transition from aquatic to terrestrial life.

## Introduction

The thylakoid membrane of *Arabidopsis thaliana* harbors the integral membrane proteins Alb3 and Alb4, which belong to the Oxa1/YidC/Alb3 protein family (later referred to as Oxa1 family). Members of this family are involved in the insertion, folding, and assembly of proteins in mitochondrial, bacterial, and thylakoid membranes (Hennon et al., 2015; McDowell et al., 2021). Alb3 plays a critical role in the insertion of the nuclear encoded light harvesting chlorophyll a/b-binding proteins (LHCPs). The LHCPs are first transported across the Toc/Tic translocon of the chloroplast envelope and then further directed to the Alb3 insertase of the thylakoid membrane *via* the posttranslational chloroplast signal recognition particle (cpSRP) pathway (Ziehe et al., 2018). After formation of a soluble LHCP/cpSRP43/cpSRP54 complex, which traverses the stroma, docking to the insertase is mediated by binding of the cpSRP targeting factors to Alb3. Direct interactions occur between Alb3 and cpSRP43 (Bals et al., 2010; Falk et al., 2010; Lewis et al., 2010; Dünschede et al., 2011) as well as between Alb3 and cpSRP54 in complex with its receptor, cpFtsY (Moore et al., 2003; Chandrasekar and Shan, 2017).

In addition to its function as a posttranslationally active insertase, Alb3 is also involved in the cotranslational biogenesis of chloroplast-encoded thylakoid membrane proteins. It is required for the cotranslational insertion of the multi-span cytochrome *b_6_f* complex subunit b_6_ (PetB), a process that involves cpSRP54 and cpFtsY and appears to be independent of the cpSec1 translocon (Kroliczewski et al., 2016). However, Alb3 is at least partially associated with the cpSec1 translocon of the thylakoid membrane suggesting a role in folding or assembly of cpSec1-inserted proteins rather than acting as insertase itself (Klostermann et al., 2002). Accordingly, it has recently been shown that a cotranslational insertion intermediate of D1, the reaction center protein of photosystem II, is associated with the cpSec1/Alb3 insertase machinery in the thylakoid membrane (Walter et al., 2015).

Consistent with a crucial role of Alb3 in thylakoid membrane protein biogenesis, the knock-out of Alb3 leads to a drastic albino phenotype in *A. thaliana* (Sundberg et al., 1997). This contrasts with the much less pronounced phenotype of *A. thaliana* plants lacking the Alb3 paralog, Alb4. These mutant plants show only a mild growth defect (Benz et al., 2009) or even no visible phenotype different from wild type (Trösch et al., 2015). However, *alb4* mutants show an impaired thylakoid structure (Gerdes et al., 2006; Benz et al., 2009). The molecular role of Alb4 is assigned to the assembly and stabilization of the ATP synthase complex (Benz et al., 2009). Furthermore, it was suggested that Alb4 participates in the biogenesis of a subset of thylakoid membrane proteins (Trösch et al., 2015; Bedard et al., 2017).

Despite the critical role of Alb3 for the biogenesis of thylakoid membrane proteins, little is known about its precise function, particularly its role in cotranslational thylakoid membrane protein biogenesis. Interestingly, it has been demonstrated that Oxa1 in the mitochondrial inner membrane and YidC paralogs in Gram-negative and Gram-positive bacteria interact directly with (translating) ribosomes during Sec-independent cotranslational membrane protein insertion. In yeast Oxa1, the matrix-exposed positively charged C-terminus plays a crucial role in the physical contact with the mitoribosome and removal of the C-terminal region strongly impairs the insertion of substrate proteins into the mitochondrial inner membrane (Jia et al., 2003; Szyrach et al., 2003). Bacterial YidC proteins (YidC in Gram-negative bacteria and the paralogs YidC1 and YidC2 in Gram-positive bacteria) also possess positively charged C-termini, which are important for ribosome binding (Wu et al., 2013; Seitl et al., 2014; Geng et al., 2015).

Here, we analyzed the ability of Alb3 and Alb4 to bind chloroplast ribosomes and show that Alb3 interacts with ribosomes involving positively charged conserved motifs in its C-terminal region, while Alb4 lacks the ability to bind ribosomes. Furthermore, we identify the ribosomal subunit uL4c as an Alb3 interaction partner and demonstrate the emergence of this interaction with the evolution of land plants.

## Materials and Methods

### Cloning, Expression, and Purification of Alb3 and Alb4 C-termini

DNA sequences coding for the C-terminal regions of Alb3 and Alb4 were cloned into the NdeI and XhoI restriction sites of the pET29b vector for expression with C-terminal His-tags using primers and cloning techniques as indicated in Supplementary Table 1. The full-length Alb3 C-terminal region [amino acids (aa) 350–462] was PCR amplified from synthesized Alb3 cDNA optimized for *Escherichia coli* codon usage (Dünschede et al., 2011) and cloned by In-Fusion cloning (Takara) following the manufacturer’s instructions. This pET29b-Alb3-C plasmid was used as template for all following deletion and truncation constructs. The plasmids pET29b-Alb3-CΔ450-462 and pET29b-Alb3-CΔ451-460 were generated utilizing the forward primer as used for pET29b-Alb3-C (see above) and adjusted reverse primers. The plasmids pET29b-Alb3-CΔ350-369, Δ370-389, Δ390-409, Δ410-429, and Δ430-449 were generated using the following cloning strategy: the complete expression plasmid leaving out the deleted region was amplified *via* PCR. The used primers contained overlapping sequences to each other enabling fusion of the PCR products by means of homologous recombination in the In-Fusion reaction (Takara) following the manufacturer’s instructions. The plasmid pET29b-Alb3CΔ397-403 was cloned using 5′ phosphorylated primers. The entire plasmid was amplified leaving out the deleted region. Afterwards the PCR product with 5′ phosphorylated ends was ligated using T4 ligase after manufacturer’s instructions (ThermoFisher Scientific) resulting in a circular plasmid. The plasmid pET29b-Alb4-C coding for the full length Alb4 C-terminus (amino acids 334–499) was generated by In-Fusion cloning (Takara) using Alb4 cDNA as template (Bals et al., 2010).

His-tagged recombinant proteins were overexpressed in *E. coli* strains BL21(DE3) or Rosetta2 (DE3) and purified under native conditions using nickel-nitrilotriacetic acid resin (Qiagen, GE Healthcare) or HisTrap^TM^ columns (GE Healthcare) and stored in 20 mM HEPES pH 8.0, 300 mM NaCl at −20°C.

### Cloning, Expression, and Purification of Full-Length Mature Alb3, Alb4, and Alb3ΔC

Alb3ΔC (aa 56–349) cDNA was PCR amplified using the pET29b-Alb3-His expression plasmid as template. This plasmid contains a codon-optimized cDNA of mature Alb3 (aa 56–462) for bacterial expression (Dünschede et al., 2011). The coding sequence of the mature Alb4 (aa 46–499) was synthesized as cDNA in an optimized form for *E. coli* codon usage by Invitrogen GeneArt. The coding sequences of Alb3ΔC and Alb4 were cloned into the NdeI/XhoI site of pET29b (Novagen) resulting in a C-terminal His_6_-tag. The sequences of the used primers are given in Supplementary Table 1.

Alb3 constructs were expressed in *E. coli* “Walker” C43(DE3) according to Dünschede et al. (2011) with the following modifications. Cells were grown in Terrific Broth (TB) medium at 37°C until an A_600_ of 0.6–0.8 was reached, induced with 1 mM isopropyl 1-thio-β-D-galactopyranoside (IPTG) and incubated for 3 h at 30°C. Harvested cells were resuspended in 3 ml of lysis buffer [50 mM Tris base, 20% (v/v) glycerol, pH 8.0] per 1 g fresh weight of the bacterial pellet for successive disruption by sonication. After centrifugation (20 min, 10,000 × *g*, 4°C) the membranes were collected from the supernatant *via* ultracentrifugation (45 min, 150,000 × *g*, 4°C) and resuspended in solubilization buffer 1 [20 mM Tris base, 20% (v/v) glycerol, 20 mM imidazole, 300 mM NaCl, pH 8] to a total volume of approximately 2 ml per liter of cell culture harvested. Zwittergent 3-12 (Merck Millipore) was added to a final concentration of 1% (w/v) and the membranes were solubilized by gentle rotation for 90 min at 4°C. The solubilized suspension was 3-fold diluted with solubilization buffer 2 (20 mM Tris base, 20 mM imidazole, 300 mM NaCl, pH 8). Insoluble material was separated from solubilized protein by centrifugation (45 min, 150,000 × *g*, 4°C). The supernatant was incubated for 90 min with Ni-NTA agarose (Qiagen), followed by two subsequent washing steps [washing buffer 1: 20 mM Tris base, 300 mM NaCl, 5% (w/v) glycerol, 20 mM imidazole, 0.3% (w/v) Zwittergent 3-12, pH 8.0; and washing buffer 2: same as washing buffer 1 but with 2% (v/v) glycerol], and the elution of Ni-NTA bound protein [elution buffer: 20 mM Tris Base, 300 mM NaCl, 250 mM imidazole, 0.3% (w/v) Zwittergent 3-12]. Imidazole was removed by either dialysis (MWCO: 10 kDa) or buffer exchange in an Amicon centrifugal filter (MWCO: 30 kDa) with storage buffer (same as elution buffer but without imidazole), after which the proteins were concentrated if necessary and either used directly or aliquoted and frozen in liquid nitrogen for storage at −80°C.

### Expression and Purification of MSP1D1

The overexpression plasmid pMSP1D1 (Denisov et al., 2004) was transformed into *E. coli* BL21(DE3). Cells were grown overnight in 2x Yeast Extract Tryptone (2xYT) medium until an A_600_ of approx. 2.5 was reached, induced with 0.3 mM IPTG and grown for an additional 4 h at 28°C. Cells were harvested by centrifugation (20 min, 3,000 × *g*) and resuspended in 10 ml of 2x TNC (40 mM Tris base, 300 mM NaCl, pH 8.0) per 1 g fresh weight of the bacterial pellet. Additionally, 1% (w/v) of Triton X-100, 12 μg/ml DNAse I and 1 cOmplete^™^ Mini protease-inhibitor tablet (Roche) per 100 ml suspension were added, followed by successive disruption by sonication. The suspension was cleared of debris by centrifugation (70 min, 15,000 × *g*, 4°C). The supernatant was supplied with 25 mM imidazole and incubated for 90 min with Ni-NTA agarose (Qiagen), followed by three subsequent washing steps (washing buffer 1: 2x TNC with 1% (w/v) Triton X-100; washing buffer 2: 2x TNC with 20 mM imidazole and 20 mM sodium cholate; and washing buffer 3: 2x TNC with 50 mM imidazole), and the elution of Ni-NTA bound protein (elution buffer: 2x TNC with 400 mM imidazole). The eluate was directly diluted with an equal part of 1x TNC. Imidazole was removed *via* dialysis (MWCO: 10 kDa) in 1x TNC, concentrated to 5 mg/ml and either used directly or aliquoted in 500 μl portions and frozen in liquid nitrogen for storage at −80°C.

### Cloning, Expression, and Purification ofHis-eGFP-uL4c Constructs

The fusions of His-eGFP to uL4 variants were constructed in two steps. First, the coding sequence of eGFP without stop codon was cloned into pETDuet (Novagen) using the restriction sites BamHI and EcoRI resulting in a His-tagged eGFP version. Second, the cDNA sequences for *A. thaliana* uL4c without its signal sequence and the ribosome-inserted loop domain (aa 150–282), as well as its C-terminal deletion construct (aa 150–262) were cloned in frame downstream of His-eGFP using the restriction sites SalI and NotI resulting in the fusions His-eGFP-uL4c and His-eGFP-uL4cΔC20. The used primers are listed in Supplementary Table 1.

The recombinant proteins were expressed in *E. coli* BL21(DE3). Cells were grown in TB medium at 37°C until an A_600_ of 0.6 was reached, induced with 1 mM IPTG and incubated for 3 h at 30°C. Harvested cells were resuspended in 5 ml of Tris lysis/wash buffer (20 mM Tris base, 300 mM NaCl, 40 mM imidazole, pH 8) per 1 g fresh weight of the bacterial pellet for successive disruption by sonication. After centrifugation, the supernatant was incubated with Ni-NTA agarose (Qiagen), followed by four washing steps with Tris lysis/wash buffer and elution with Tris elution buffer (20 mM Tris base, 300 mM NaCl, 250 mM imidazole, pH 8). Eluates were desalted with a PD-10 column (GE Healthcare) with storage buffer (20 mM Tris base, 300 mM NaCl, pH 8), after which the proteins were concentrated if necessary and either used directly or aliquoted and frozen in liquid nitrogen for storage at −80°C.

### Construction of Plasmids and Subsequent Alkaline Phosphatase Assay

Coding sequences of precursor and mature alkaline phosphatase (pAP and mAP, respectively) were amplified from *E. coli* genomic DNA and cloned into the pET29b vector using NdeI and XhoI restriction sites resulting in a C-terminal His-Tag. Fusion constructs were generated based on the pET29b-Alb3-His expression vector (Dünschede et al., 2011). In a first step a restriction site (SalI) was introduced at the planned insertion site for mAP in the pET29b-mAlb3-His plasmid using QuikChange mutagenesis (Agilent). Afterwards, the plasmid was cut at this restriction site and the PCR product encoding mAP was inserted with linker sequences (three glycines on each site) using the In-Fusion cloning technique (Takara). The mAP fusion to the C-Terminus of Alb3 was generated *via* In-Fusion cloning after XhoI restriction of pET29b-Alb3-His. The mAP PCR product was inserted at Alb3’s C-terminal end. The used primers are listed in Supplementary Table 1.

The pET29b expression plasmids of the different constructs were transformed into *E. coli* Rosetta gami2 (DE3), which lacks the endogenous alkaline phosphatase (ΔAP). The strains were grown under selective pressure in LB liquid culture at 37°C overnight. The activity of the alkaline phosphatase was analyzed according to Feng et al. (2011) with some minor modifications. Droplets of 20 μl of the overnight cultures were pipetted onto LB agar plates containing kanamycin for the selective pressure, 1 mM IPTG for induction of mAlb3-mAP fusion protein expression and 0.4 mg/ml 5-bromo-4-chloro-3-indolyl phosphate (BCIP) as a substrate for the alkaline phosphatase. The bacterial plates were incubated at 37°C overnight. Afterwards, the blue staining of the bacterial droplets was analyzed.

### Isolation of Chloroplast 70S Ribosomes and Sucrose Density Gradient Centrifugation

Chloroplast ribosomes were isolated from 9-day-old pea plants as described previously (Hristou et al., 2019). Sucrose density gradient centrifugation was conducted with 1 nmol of the indicated recombinant His-tagged protein constructs or 1 nmol of nanodisc-reconstituted protein or empty nanodiscs and enriched chloroplast ribosomes (1.2 mg) in LSB (50 mM potassium acetate, 20 mM HEPES-KOH pH 7.5, 6 mM magnesium acetate, 2 mM dithiothreitol) as described previously (Hristou et al., 2019). Fractions of the gradients were analyzed by immunoblots.

### Preparation of Nanodiscs

For the preparation of MSP1D1-asolectin NDs with and without inserted membrane protein, all reagent stocks were solubilized in standard ND-buffer [20 mM Tris base, 300 mM NaCl, 0.5 mM ethylenediaminetetraacetic acid (EDTA), pH 8.0] and pipetted on ice. The assembly mix followed the molar stochiometry 0.1:1:65:195 [membrane protein:MSP1D1:asolectin (Merck):n-dodecyl-β-D-maltoside (β-DDM)], omitting the membrane protein in the case of empty MSP1D1-asolectin NDs. The mixtures were gently rotated at 4°C for 1 h and subsequently incubated with Bio-Beads SM-2(Bio-Rad) according to the manufacturer’s instructions at 4°C for 2 h. The Bio-Beads were removed using a spin filter. If necessary, the samples were concentrated to a volume smaller or equal to 500 μl prior to being fractionated on a Superdex 200 10/300 GL column (GE Healthcare) equilibrated with ND-buffer using an ÄKTA FPLC-system (GE Healthcare). Empty MSP1D1 NDs elute at approximately 13.0 ml and ND-reconstituted Alb3, Alb4 and Alb3ΔC at approximately 10–10.5 ml (Supplementary Figure S1). The collected ND fractions were concentrated to approximately 1 nmol inserted protein (or MSP1D1 with empty NDs) per 100 μl and either used directly or aliquoted in 100 μl portions and frozen in liquid nitrogen for storage at −80°C. A successful reconstitution in NDs was determined from the comigration behaviour of inserted protein and MSP1D1 after application onto the gel filtration column and the presence of asolectin lipids in ND fractions, as determined by thin layer chromatography (Supplementary Figure S1). A negative control reconstitution reaction conducted in the absence of MSP1D1 resulted in complete precipitation of Alb3 during the detergent removal step (data not shown).

### Construction of Split-Ubiquitin Plasmids and the Split-Ubiquitin Assay

The construct pAMBV4-Alb3 was previously described (Pasch et al., 2005). Cyanobacterial cDNAs used in this method were sourced from genomic DNA and amplified with the same primers used for the later cloning into pADSL-Nx. The cDNAs coding for *A. thaliana* mature uL4c (aa 50–282) and constructs thereof (uL4cΔC20: aa 50–262; uL4cΔN231: aa 232–282) were cloned into the BamHI/XhoI site of pADSL-Nx. Full-length *Synechocystis* sp. *PCC6803* uL4 (aa 1–210), full-length *Thermosynechococcus elongatus* uL4 (aa 1–210) and constructs thereof (uL4+C20: fusions of the cyanobacterial full-length uL4 proteins and *A. thaliana* uL4c aa 263–282) were cloned into the SalI/BglII site of pADSL-Nx. The C+20 fusion constructs were generated by 2-fold consecutive amplification with extended reverse primers. The cDNA encoding mature Alb4 (aa 46–499) was cloned into the XbaI/StuI site of pAMBV4. Sequences of the used primers are indicated in Supplementary Table 1. The split-ubiquitin assay was done as described previously (Pasch et al., 2005).

### Size Exclusion Chromatography Interaction Assays

Analytical size exclusion chromatography experiments were performed with an ÄKTA purifier system (GE Healthcare), using a Superdex 200 10/300 GL column (GE Healthcare). The column was equilibrated in ND-buffer, and the sample injection volume was 500 μl and the flow rate was 0.4 ml/min. A total of 2 nmol (or ND-equivalents) of each of the indicated tested proteins were combined and filled up to 500 μl with ND-buffer and incubated for 30 min on ice prior to application. The fractioned samples were analyzed by immunoblot analyses.

### Immunoblot Analyses

Proteins were blotted from SDS-polyacrylamide gels onto nitrocellulose membrane (Macherey-Nagel). Transferred proteins were detected using antibodies against the His-tag (Penta His HRP Conjugate, Qiagen, dilution 1:4,000) and chloroplast uL4 (Agrisera, dilution 1:5,000), *A. thaliana* Alb3 (α-Alb3: Bals et al., 2010, dilution 1:500), MSP1D1 (α-ApoA1, Sigma-Aldrich, dilution 1:2,000), eGFP (α-eGFP, Sigma-Aldrich, dilution 1:5,000).

### Sequence Search, Alignment, and Tree Construction of Plastid and Cytosolic uL4 Proteins

By using the *A. thaliana* uL4c peptide sequence as a template, a pBLAST-search of multiple genome archives and subsequent manual refinement of the results according to likelihood and sequence databank annotation provided an overview of chloroplast, rhodoplast, and cyanobacterial uL4 sequences (see Supplementary File 1). The validity of the chloroplastic peptide sequences was further evaluated *via* ChloroP 1.1 analysis, determining whether the chosen sequences possess a chloroplast signal peptide (Emanuelsson et al., 1999). The cytosolic uL4 sequences were retrieved from the same databases in accordance with the selected green algal and streptophyte sequences to provide a specific comparison with the chloroplast sequences. Finally, for subsequent cross species phylogenetic analysis, the N-terminal signal peptide and gross outlier sequences were removed to focus on the homologous regions.

In total, 98 amino acid sequences from 69 different species were aligned with MAFFT v7.471 (Katoh and Standley, 2013) using the L-INS-i algorithm. A total of 548 positions were in the final dataset. The alignment was visualized in JalView (Waterhouse et al., 2009). Phylogenetic analysis was conducted in MEGA X (Kumar et al., 2018; Stecher et al., 2020). The evolutionary history was inferred using the Maximum Likelihood method and JTT matrix-based model (Jones et al., 1992) with 1,000 bootstrap replicates. The tree with the highest log likelihood was exported in Newick format (see Supplementary File 2) and subsequently used for unrooted tree visualization and phylogenetic analysis in SplitsTree4 v.4.15.1 (Huson and Bryant, 2006).

## Results

### Assignment of the C-terminal Region and Protein-Binding Sites of Alb3 Using the Alphafold2 Structural Model

**Figure 1:**
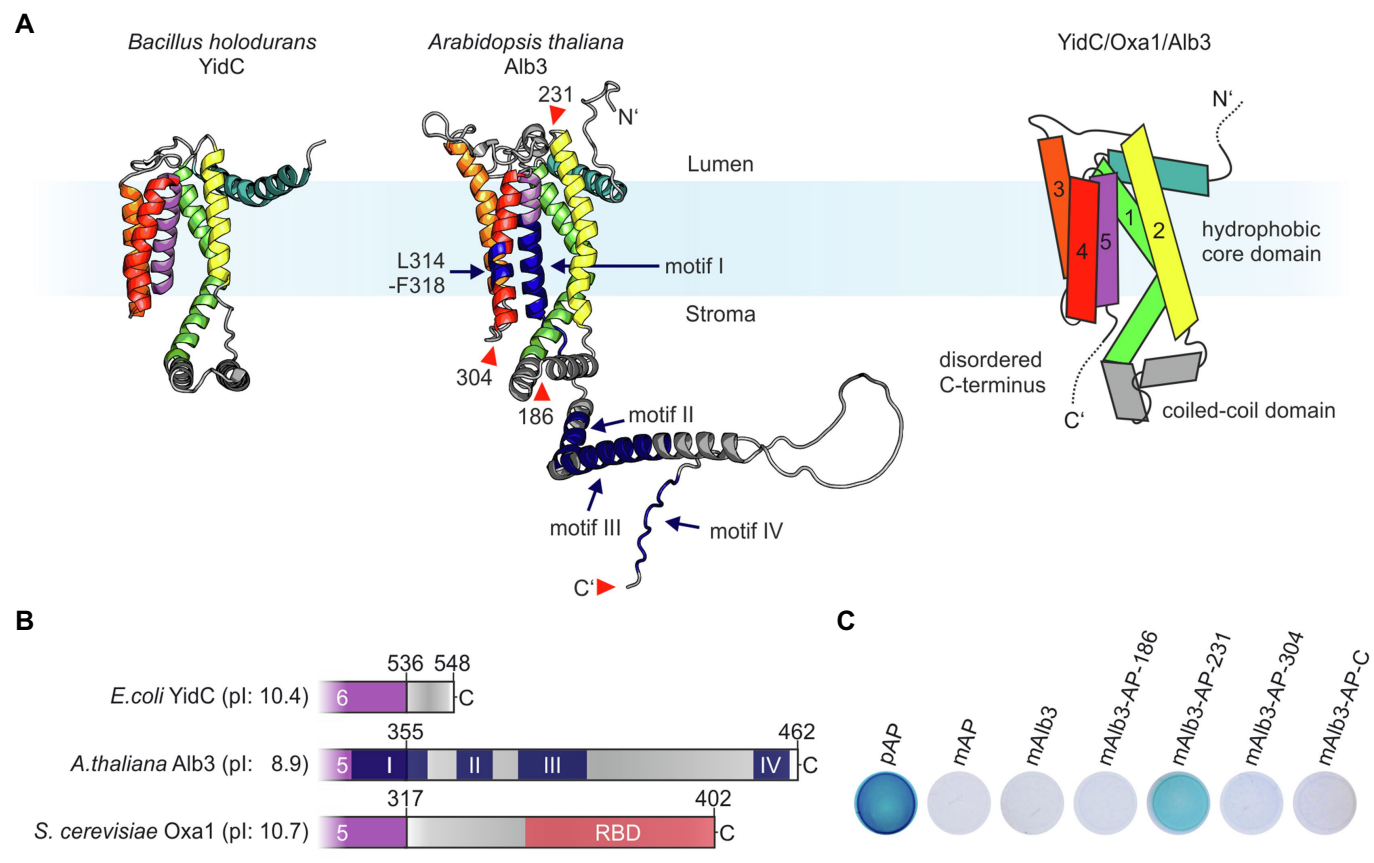
Proteins of the Oxa1 family share common structural features. **(A)** Comparison of the crystal structure of YidC from *Bacillus halodurans* (left; PDB: 3WO6; Kumazaki et al., 2014a) and the AlphaFold model of *Arabidopsis thaliana* Alb3 without signal peptide (middle; model: AF-Q8LBP4-F1; Jumper et al., 2021). Regions in the structural model of Alb3 highlighted in dark blue indicate conserved sequence motifs involved in cpSRP43 binding (residues 314–318 within the fourth TMH and motifs II and IV in the C-terminal region); the conserved motifs I and III are also highlighted in dark blue. The red arrowheads indicate the alkaline phosphatase (AP) insertion sites mentioned in **C**. Right: scheme of the predicted topology of the hydrophobic core region of members of the Oxa1 family encompassing five transmembrane helices. **(B)** Features of the C-terminal sequences of members of the Oxa1 family. The start of the C-terminal regions were defined to the first residue after the last transmembrane helix according to the crystal structure of *Escherichia coli* YidC (PDB: 3WVF; Kumazaki et al., 2014b) and the Alphafold models of *A. thaliana* Alb3 (see above) and *Saccharomyces cerevisiae* Oxa1 (model: AF-P39952-F1; Jumper et al., 2021). Theoretical pI-values for the indicated C-terminal peptide sequences were calculated using ExPASy ProtParam. Prominent sequence motifs are marked. I, II, III, and IV: conserved motifs of the Alb3 C-terminal region (Falk et al., 2010); RBD: conserved helical ribosome binding domain of the Oxa1 C-terminal region (Jia et al., 2003). **(C)** Alkaline phosphatase (AP) activity of bacterial cells carrying expression plasmids coding for different Alb3-AP fusions to identify membrane protein topology. AP was fused into mature Alb3 at amino acid positions 186 (mAlb3-AP-186), 231 (mAlb3-AP-231), 304 (mAlb3-AP-304), and to the C-terminus (mAlb3-AP-C). Control cells expressed the precursor of AP (pAP), mature AP (mAP) and mAlb3. The positions of the fusions are indicated schematically in **A** (red arrowheads).

In 2014, the first high-resolution structures of members of the Oxa1 family, YidC from *Bacillus halodurans* and *E. coli*, were published providing insight into the mechanistic function of these types of insertases (Kumazaki et al., 2014a,b). Subsequently, structural analyses by crystallization or cryo-electron microscopy revealed the architecture of *Thermotoga maritima* YidC and ribosome-bound *E. coli* YidC (Wickles et al., 2014; Xin et al., 2018). These studies showed that YidC insertases are characterized by a conserved hydrophobic core region comprising five transmembrane helices (TMHs), which form a membrane-embedded hydrophilic groove mediating protein insertion. They also share a conserved coiled-coil domain between the first two TMHs of the hydrophobic core and a positively charged extension at their C-terminus (Figures 1A,B). Currently, no comparable structural data are available for plastidic Alb3 proteins. We therefore used the structure prediction from the EMBL-EBI-AlphaFold database (Jumper et al., 2021) to define the positions of the transmembrane helices and soluble domains in Alb3. As expected, Alb3 shows the conserved overall architecture of YidC insertases comprising five TMHs (Figure 1A). The model indicates that the low-structured positively charged C-terminal extension starts at amino acid position 355, encompassing 107 residues. Not only an extended C-terminal region is absent in *E. coli* YidC but is also characteristic of mitochondrial Oxa1 proteins (Figure 1B). Four conserved, positively charged sequence motifs have been discussed for Alb3 proteins (Falk et al., 2010). The Alb3 C-terminal domain contains the positively charged motifs II to IV. Motifs II and IV have been shown to be involved in binding cpSRP43 (Falk et al., 2010; Dünschede et al., 2011; Horn et al., 2015; Liang et al., 2016) and the cpSRP54/cpFtsY complex (Chandrasekar and Shan, 2017). Motif I described by Falk et al. (2010) is largely assigned to the alpha-helical region of the fifth TMH. Notably, the positions of the TMHs in the structural AlphaFold-model differ significantly from previous predictions based on hydrophobicity plots. Accordingly, the previously described membrane-embedded cpSRP43-binding site mapped to residues 314–318 (Dünschede et al., 2011) is located in the fourth TMH at a position close to the stromal opening of the hydrophilic grove in the new model (Figure 1A). To support the membrane topology of Alb3, the alkaline phosphatase (AP) fusion approach in *E. coli* was applied, which is based on the observation that alkaline phosphatase is enzymatically active only when localized in the periplasm (Manoil and Beckwith, 1986). To this end, mature AP was fused into the first and second stromal loops (positions 186 and 304), the first lumenal loop (position 231) and to the C-terminus. The resulting Alb3 fusions were expressed in *E. coli* and the AP activity determined (Figure 1C). Bacterial cells expressing full-length AP, including the precursor sequence, that codes for secretion of AP into the periplasmic space, mature AP, or mature Alb3 were used as controls. Significant AP activity was only observed for the lumenal loop fusion construct while all other fusion constructs showed no or very low activity. These data indicate that the first lumenal loop is directed into the periplasm, while the stromal loops and the C-terminus have a cytosolic orientation when Alb3 is expressed in bacteria and are consistent with the predicted topology of Alb3.

### The C-terminal Region of Alb3 but not of Alb4 Interacts with Ribosomes

**Figure 2:**
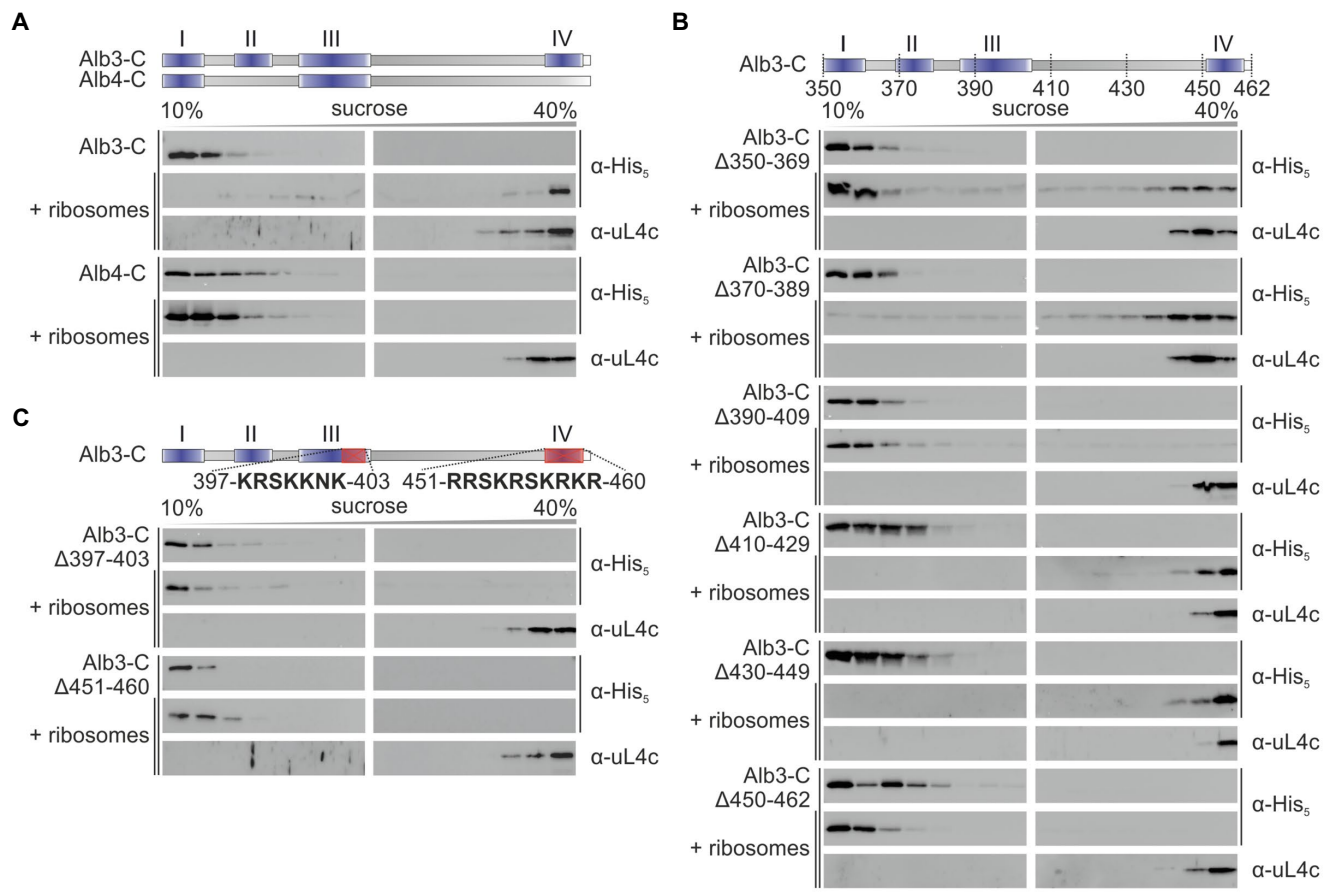
Sucrose gradient interaction assay of recombinant Alb3 and Alb4 C-terminus variants with chloroplast ribosomes. The recombinant His-tagged C-terminal regions of Alb3 and Alb4 and the indicated deletion constructs were incubated with chloroplast ribosomes and loaded onto a sucrose density gradient. After ultracentrifugation, the gradient fractions were analyzed immunologically using antibodies against the His-tag (α-His) and the chloroplast ribosomal protein uL4c (α-uL4c). Sucrose density gradient centrifugation of the recombinant proteins in the absence of ribosomes was used as negative control. Alb3-C, C-terminal region of Alb3 (amino acids 350–462); Alb4-C, C-terminal region of Alb4 (amino acids 334–499). **(A)** Comparison of Alb3-C and Alb4-C. **(B)** A successive experimental series of 20 amino acid deletions introduced into the Alb3-C construct. **(C)** Targeted deletions of highly conserved arginine- and lysine-rich sequences within motif III and motif IV.

To analyze whether the C-terminal region of Alb3 interacts directly with the chloroplast ribosome a recombinant C-terminally His-tagged construct comprising amino acid residues 350–462 (Alb3-C) was co-fractionated with *Pisum sativum* chloroplast ribosomes on sucrose density gradients. The sedimentation pattern of Alb3-C and the ribosomes were analyzed by immunoblot analysis of the sucrose gradient fractions using antibodies directed against the His-tag and the ribosomal subunit uL4, respectively (Figure 2A). While in absence of ribosomes Alb3-C stayed in the top of the gradient, a clear migration of Alb3-C to the bottom of the gradient was detected in presence of ribosomes. To analyze whether the ribosome binding ability is specific for Alb3, analogous experiments were conducted with the recombinant C-terminal region of the paralog Alb4, which lacks motifs II and IV (Falk et al., 2010; Figure 2A). This region comprises residues 334–499. Notably, no cofractionation was observed for Alb4-C and ribosomes (Figure 2A). To identify regions of Alb3-C involved in ribosome binding sequential deletion of 20 amino acid residues were introduced into Alb3-C starting at position 350 (Figure 2B). The Alb3-C variants Alb3-CΔ390-409 and Alb3-CΔ450-462 showed no comigration with ribosomes in sucrose gradients while all other deletion constructs (Alb3-CΔ350-369, Alb3-CΔ370-389, Alb3-CΔ410-429, and Alb3-CΔ430-449) were not impaired in their ability to bind ribosomes. Interestingly, the Alb3 region comprising amino acids 450–462 represents the extreme C-terminus of Alb3 containing the positively charged binding motif IV. The Alb3 region 390–409 harbors the conserved motif III that is also enriched in positively charged residues, but its function has not yet been assigned. To analyze whether the positively charged clusters of motif IV (451-RRSKRSKRKR-460) and motif III (397-KRSKKNK-403) are important for ribosome binding, the corresponding Alb3-C deletion constructs were generated. As shown in Figure 2C, both constructs were not able to bind ribosomes. Therefore, our data suggest that motif III and motif IV are cooperatively required for ribosome binding. In line with this, the inability of Alb4 to interact with ribosomes correlates with the absence of motif IV in Alb4.

### Besides the C-terminal Region, Alb3 Provides an Additional Binding Site for Ribosomes

**Figure 3:**
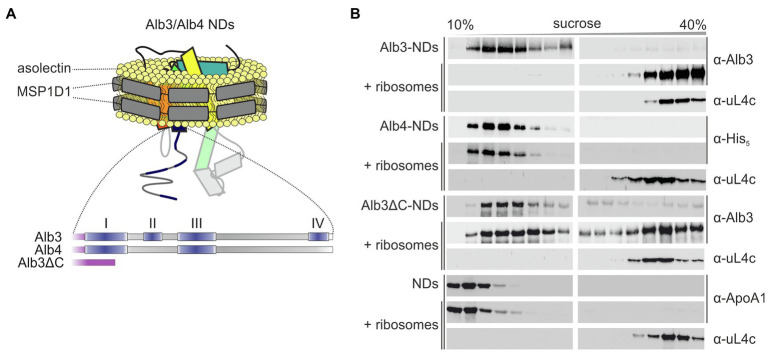
Sucrose gradient interaction assay of Alb3 variants and Alb4 reconstituted in nanodiscs with chloroplast ribosomes. **(A)** Schematic representation of recombinant His-tagged full-length mature Alb3 (amino acids 55–462), Alb3ΔC (amino acids 55–369) and Alb4 (amino acids 46–499) reconstituted in MSP1D1 nanodiscs (NDs; not to scale) with asolectin as bilayer forming lipid. **(B)** Nanodiscs (NDs) containing Alb3 (Alb3-NDs), Alb4 (Alb4-NDs), or Alb3ΔC (Alb3ΔC-NDs) and NDs without inserted membrane protein as negative control were incubated with chloroplast ribosomes and loaded onto a sucrose density gradient. After ultracentrifugation, the gradient fractions were analyzed immunologically using antibodies against the His-tag (α-His), *A. thaliana* chloroplast Alb3 (α-Alb3), human Apolipoprotein A1 (α-ApoA1; the wildtype template of MSP1D1) and the chloroplast ribosomal protein uL4c (α-uL4c). Sucrose density gradient centrifugation of the Alb constructs, containing NDs in the absence of ribosomes was used as negative control.

To determine whether the C-terminal region of Alb3 is the sole binding platform for ribosomes, the detergent-solubilized recombinant mature full-length Alb3 and a variant lacking the C-terminal residues 350–462 (Alb3∆C) were reconstituted into nanodiscs (NDs), composed of soybean asolectin and the scaffold protein MSP1D1 (Figure 3A and Supplementary Figure S1) and subjected to sucrose gradient centrifugation in the presence and absence of pea chloroplast ribosomes.

As expected, Alb3-NDs comigrated with ribosomes to the bottom of the gradient, while Alb3-NDs stayed in the upper part of the gradient in absence of ribosomes (Figure 3B). A control reaction showed that the comigration is not due to an interaction between the NDs themselves and ribosomes, as empty NDs stayed at the top of the gradient in the presence of ribosomes (Figure 3B). A comigration with ribosomes to the bottom of the gradient was also detected for the Alb3∆C-NDs, suggesting that Alb3 has at least one additional contact site for ribosomes within the core region. Since the mature Alb3 and Alb4 protein sequences have an identity of about 72%, it was reasonable to assume that this ribosome binding site might be conserved in Alb4. To analyze whether mature full-length Alb4 binds ribosomes, sucrose gradient centrifugations in absence and presence of ribosomes were conducted using Alb4-NDs. Notably, no comigration was detected between Alb4-NDs and ribosomes confirming that Alb4 is not a ribosomal interaction partner (Figure 3B).

### Alb3 Interacts with the Negatively Charged C-terminal Region of the Chloroplast Ribosomal Subunit uL4c

**Figure 4:**
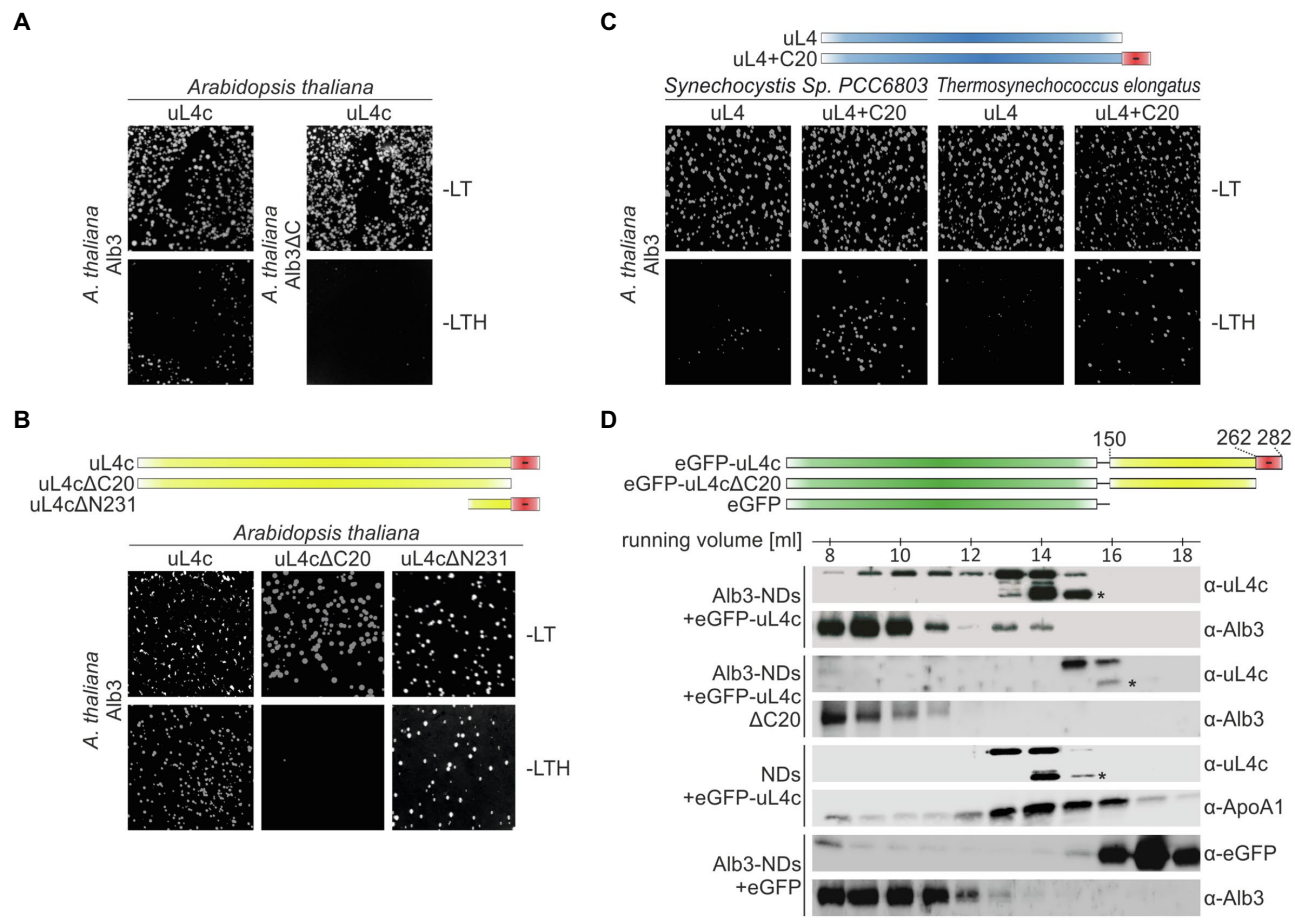
Split ubiquitin and size exclusion chromatography interaction assays between Alb3 and uL4c constructs. Split ubiquitin assays **(A–C)** were performed employing fusions to the C- (Cub) and N- (NubG) terminal halves of ubiquitin. The Alb3 constructs were C-terminally fused to Cub and the uL4 constructs were N-terminally fused to NubG. Alg5-NubI and Alg5-NubG served as positive and negative controls, respectively. Yeast colonies were plated on permissive medium (-LT) and on selective medium (-LTH). A full version with all control experiments is shown in Supplementary Figure S2. **(A)** Comparison between the interaction of mature Alb3 (amino acids 55–462) and C-terminally truncated Alb3 (amino acids 55–369; Alb3ΔC) with *A. thaliana* uL4c. **(B)** Interaction assay between Alb3 and uL4c or uL4c deletion constructs lacking 20 C-terminal amino acids (uL4cΔC20) or 231 N-terminal amino acids resulting in a construct coding for the last 50 amino acids (uL4cΔN231). **(C)** Interaction assay between Alb3 and cyanobacterial uL4 proteins or fusions of cyanobacterial uL4 proteins with the C-terminal 20 amino acids of *A. thaliana* uL4c (uL4+C20). **(D)** Size exclusion chromatography assays were performed by incubating Alb3-NDs with fusion constructs of eGFP and the C-terminal half of *A. thaliana* uL4c with and without its negative C-terminus, eGFP-uL4c (amino acids 150–282) and eGFP-uL4cΔC20 (amino acids150–262), respectively. Control assays were conducted using Alb3-NDs in combination with eGFP and empty NDs in combination with eGFP-uL4c. The elution fractions between 7.5 and 18.5 ml were analyzed immunologically using antibodies against *A. thaliana* chloroplast Alb3 (α-Alb3), *A. thaliana* chloroplast ribosomal protein uL4c (α-uL4c), Human Apolipoprotein A1 (α-ApoA1; the wildtype template of MSP1D1) and eGFP (α-GFP). Asterisks mark C-terminal degradation bands of the eGFP-uL4c-constructs.

**Figure 5:**
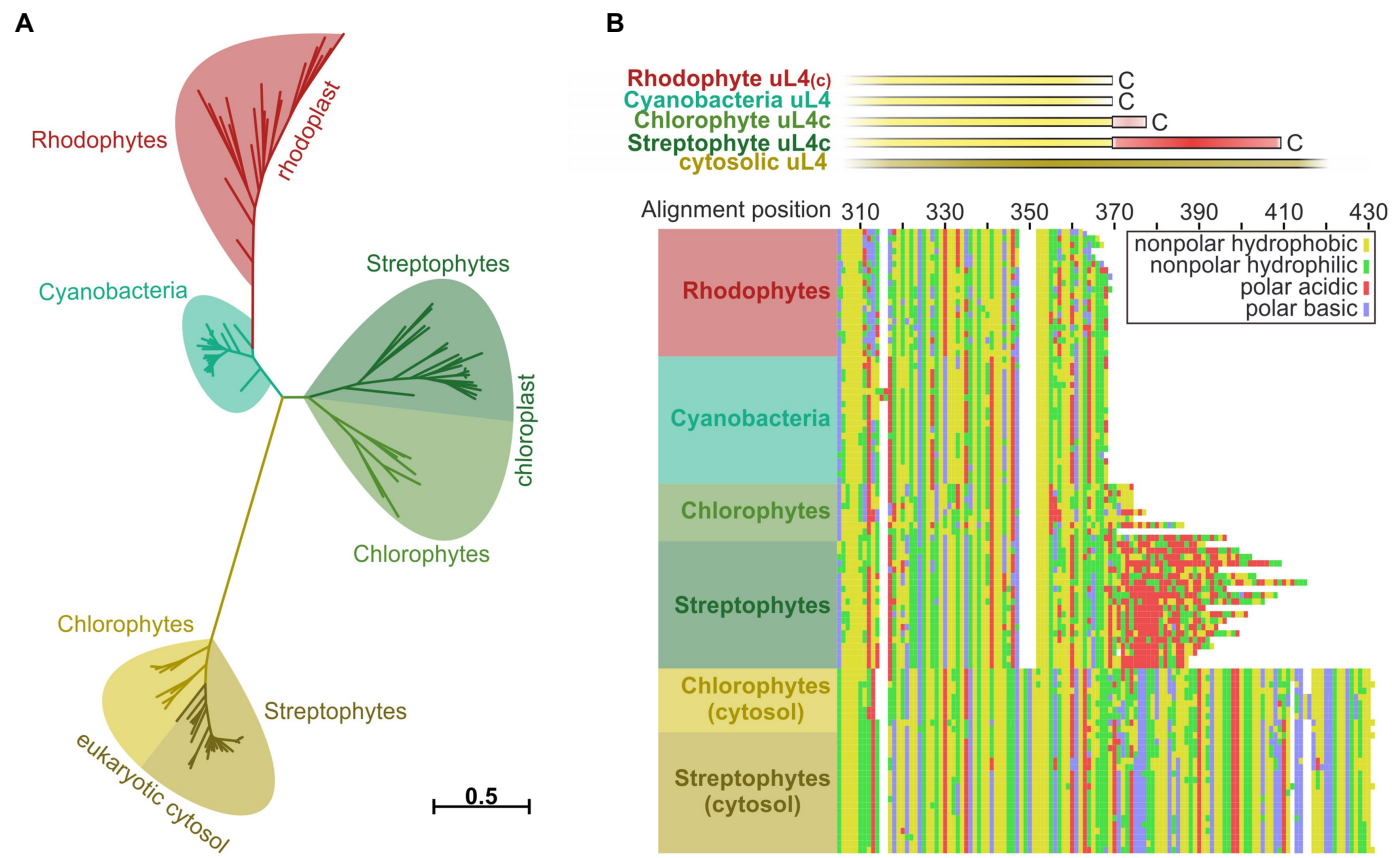
Reconstruction of phylogenetic relationships and amino acid sequence comparisons between uL4 proteins of photosynthetic organisms. Phylogeny- and multiple sequence alignment-based analysis of plastidic and cytosolic uL4 sequences from chlorophytes, streptophytes, rhodophytes, and cyanobacteria. **(A)** Unrooted maximum likelihood tree of the homologous uL4 peptide sequence regions. Branch lengths are scaled according to the number of substitutions per site. **(B)** MAFFT alignment of the uL4 amino acid sequences, using the L-INS-I algorithm. Top: schematic representation of the aligned sequences. Bottom: Cropped alignment emphasizing the C-terminal regions of the bacterial/plastidic uL4/uL4c proteins. Amino acids are represented by their chemical properties: non-polar hydrophobic (yellow; A,F,I,L,M,P,V,W), non-polar hydrophilic (green; C,G,N,Q,S,T,Y), polar basic (blue; H,K,R) and polar acidic (red; D,E).

The conserved positively charged clusters of motif III and motif IV of Alb3 show similarity to the ARRKR motif in the C-terminal tail region of cpSRP54, which was previously shown to interact with cpSRP43 and to be involved in binding the ribosomal subunit uL4c in cotranslational targeting (Funke et al., 2005; Hristou et al., 2019). We therefore considered uL4c as a potential ribosomal binding partner of the Alb3 C-terminus. To test this hypothesis the yeast split-ubiquitin two-hybrid system was employed. Yeast cells were cotransformed with two plasmids encoding Alb3 or Alb3ΔC fused to the C-terminal half of ubiquitin (Cub) and uL4c fused to a point mutant of the N-terminal half of ubiquitin (NubG). As shown in Figure 4A and Supplementary Figure S2, Alb3 indeed interacted with uL4c in the split-ubiquitin assay and removal of the Alb3 C-terminal region abolished this interaction. Strikingly, streptophytic uL4c is characterized by a highly negatively charged C-terminal extension (Figure 5) leading to the hypothesis that the positively charged Alb3 C-terminal region might bind to this negative patch of uL4c. To test this hypothesis, an uL4c variant lacking the last 20 residues at the C-terminus (uL4c∆C20) and a variant consisting only of the last 50 C-terminal residues (uL4c∆N231) were used in the split-ubiquitin system. While uL4c∆C20 did not interact with Alb3, uL4c∆N231 showed a clear interaction (Figure 4B and Supplementary Figure S2). These data strongly suggest that a negatively charged patch of 20 amino acids at the C-terminus of uL4c mediates binding to Alb3. This result is corroborated by the finding that the uL4 proteins of *Synechocystis* sp. *PCC 6803* and *T. elongatus*, lacking a negatively charged C-terminal extension showed no interaction with Alb3, while fusions of these cyanobacterial uL4 proteins and the C-terminal 20 amino acids of *A. thaliana* uL4c clearly interacted with Alb3 (Figure 4C and Supplementary Figure S2).

To further confirm the interaction between Alb3 and the C-terminal region of uL4c using recombinant proteins, soluble fusions of eGFP and the C-terminal globular domain of uL4c (residues 150–282; eGFP-uL4c) and the same construct lacking the C-terminal 20 amino acids of uL4c (eGFP-uL4c∆C20) were generated. Both constructs lack the N-terminal part of uL4c harboring the extended finger loop that forms part of the ribosomal peptide tunnel. This approach was chosen as recombinant full-length uL4c and shorter constructs without eGFP fusion tended to aggregate (data not shown). The recombinant uL4c constructs were incubated with Alb3-NDs followed by fractionation using size exclusion chromatography. Western blot analyses of the collected fractions using antibodies against uL4c and Alb3 showed that eGFP-uL4c partially comigrated to the lower elution volumes together with the significantly larger Alb3-NDs (Figure 4D). No comigration was detected between eGFP-uL4c∆C20 and Alb3-NDs. Negative controls using eGFP-uL4c and empty NDs or eGFP and Alb3-NDs showed no shift of eGFP-uL4c or eGFP to higher molecular weight fractions (Figure 4D). These data reinforce the importance of the uL4c C-terminus for binding Alb3.

### The Negatively Charged C-terminus of uL4c Evolved During the Transition From Aquatic to Terrestrial Life

Several components and interaction sites of the posttranslational and cotranslational SRP pathways evolved and changed during the transition from prokaryotic to eukaryotic photosynthetic organisms and from water-based to land-based plant life (Ziehe et al., 2017; Hristou et al., 2019). To evaluate the phylogeny of the ribosomal subunit uL4c, a phylogenetic analysis of plastid and cytosolic uL4 amino acid sequences from chlorophytes, streptophytes, rhodophytes, and cyanobacteria was performed. We expected a comparably high degree of conservation within the ribosomal proteins of photosynthetic prokaryotes and related cell organelles in accordance with the endosymbiotic theory. Consistently, the plant cytosolic uL4 proteins form an outgroup of eukaryotic origin with large unaligned insertions and an extension by comparison to the remainder of the sequences (Figure 5B; Supplementary Figure S4). The evolutionary distance among the remaining groups of proteins was considerably shorter (Figure 5A; Supplementary Figure S3). The larger degree of amino acid sequence conservation within the cyanobacterial/organellar proteins is also visible in the alignment, when compared to the eukaryotic cytosolic uL4 proteins (Figure 5B; Supplementary Figure S4). Interestingly, the negatively charged C-terminal extension of the chloroplast uL4c seems to be characteristic of streptophytic species including charophytes (*Chara braunii*), bryophytes (e.g., *Physcomitrium patens*), lycophytes (e.g., *Selaginella moellendorffii*), monilophytes (e.g., *Azolla filiculoides*), and spermatophytes (e.g., *A. thaliana*). These data suggest that the negatively charged C-terminal extension of the chloroplast uL4c evolved as a molecular adaptation to life on land. The C-terminal extension shows no discernible degree of strict sequential interspecies conservation, hinting that only the charge itself was conserved but not necessarily the structure. A disorder analysis (online disorder prediction tool PrDOS; Ishida and Kinoshita, 2007) of *P. sativum* uL4c shows this C-terminal region to be highly disordered, and according to current structural models of the chloroplast ribosome it is likely positioned at the ribosomal surface (Supplementary Figure S5A).

## Discussion

The cotranslational insertion of membrane proteins requires a tight coupling of the ribosome to the insertase in the target membrane to enable an efficient transfer of the nascent chain into the protein-conducting channel of the insertase. A physical association between ribosomes and mitochondrial Oxa1, bacterial YidC, and the Sec machinery in the endoplasmatic reticulum or bacterial plasma membrane have been demonstrated biochemically and by structural analyses using cryo-EM techniques (Jia et al., 2003; Szyrach et al., 2003; Kohler et al., 2009; Frauenfeld et al., 2011; Kedrov et al., 2013; Wu et al., 2013; Seitl et al., 2014; Wickles et al., 2014; Kater et al., 2019; Gemmer and Forster, 2020; Itoh et al., 2021). However, the tethering mechanism of chloroplast ribosomes to insertases of the thylakoid membrane remained unexplored to date. In this study, we show that the stromally exposed C-terminus of the membrane insertase Alb3 interacts with chloroplast ribosomes and that the positively charged motifs III and IV of Alb3 are involved in ribosome binding. These results are consistent with the finding that the positively charged C-termini of YidC and Oxa1 contribute to cotranslational ribosome tethering (Jia et al., 2003; Szyrach et al., 2003; Seitl et al., 2014; Geng et al., 2015). Additionally, we show that the C-terminal region of Alb3 is not the only ribosome binding site within Alb3. It was demonstrated that YidC contacts the ribosome with two additional points, the two cytosolic loop regions C1 and C2 (Wickles et al., 2014), whereby the deletion of C2 but not of C1 had a direct impact on ribosome binding (Geng et al., 2015). Based on our results, we speculate that homologous regions in Alb3 contribute to ribosome binding in addition to the C-terminus. An interaction of the ribosome with both the core domain and the C-terminal extension was also described for human Oxa1 (Itoh et al., 2021).

Interestingly, we identified the ribosomal protein uL4c as an interaction partner of Alb3 and demonstrate that the C-terminal region of uL4 mediates this interaction. This region is highly unstructured, negatively charged and likely exposed on the surface of the ribosome (Supplementary Figure S5A). In the ribosome, uL4c is located away from the exit site of the ribosomal polypeptide tunnel, which makes this interaction unexpected in the light of previous studies, which have mapped the ribosome binding interfaces of members of the Oxa1 family: YidC has been shown to interact with the ribosomal tRNA helix H59 and the subunits uL24, uL29 and uL23, all of which are part of the ribosomal exit site or at least in close proximity (Kedrov et al., 2016). Oxa1 was shown to cross-link with uL23m and uL24m (Jia et al., 2009), both of which line the exit of the ribosomal peptide tunnel. Recently, an additional contact site between the extreme C-terminus of Oxa1 and the ribosomal subunits bL28 and uL29 was discovered in a cryo-electron microscopic structure of a human Oxa1/mitoribosome complex. This site is located at a greater distance from the membrane surface (about 70 Å) but is still close to the tunnel exit (Itoh et al., 2021), as uL29 forms a part of the exit rim. Interestingly, it was recently shown that uL4c also interacts with chloroplast SRP54 (cpSRP54) *via* a conserved Lys/Arg-rich motif in its C-terminal region, which is similar to motif IV of Alb3 (Hristou et al., 2019). As cpSRP54 in complex with its receptor, cpFtsY, binds to Alb3 during cotranslational protein transport, we speculate that this interaction is part of the transfer of uL4c to Alb3 to thermodynamically favor the release of cpSRP54 by competing with cpSRP54 for uL4 binding and to establish correct ribosome positioning on the membrane. Since our data show that removal of the C-terminus of Alb3 prevents interaction with uL4 but not binding to ribosomes, it is reasonable to assume that the core domain of Alb3 associates with additional ribosomal components possibly located at the exit of the peptide tunnel. In this case, it is likely that Alb3 acts as an insertase itself in cotranslational transport. However, our knowledge about this insertion mechanism is very limited. So far, only PetB, a cytochrome *b_6_f* component, was shown to be inserted cotranslationally by Alb3 itself (Kroliczewski et al., 2016).

The C-terminal regions of Alb3 and uL4c are predicted to be inherently disordered (Falk et al., 2010; Supplementary Figure S5B) and contain positively charged conserved motifs and negatively charged patches giving them a positive or negative charge bias, respectively. Such disordered regions have the potential to bind multiple different substrates due to a high accessibility of all binding motifs within their sequences and allow the interaction over a larger surface with their binding partners (Wright and Dyson, 2015). Additionally, electrostatic interactions were shown to play a crucial role in longer-range interactions in proteins, remaining strong even at a distance of 5–10 Å (Zhou and Pang, 2018). The Alb3/ribosome interaction could therefore be initiated from a relatively large distance to allow efficient processing of ribosome nascent chains during cotranslational membrane insertion. The size of the Alb3 C-terminus and the presence of ribosomal interaction sites within the C-terminus and the core domain may indicate a high degree of spatial control for ribosome binding. The limitation of mobility during cotranslational insertion might assist in the assembly of larger transmembrane complexes such as the photosystems 1 and 2 by coordinating the interaction of the ribosome with assembly factors, as was observed for Oxa1 (Keil et al., 2012).

However, it should be noted that we used a heterogeneous stromal ribosome population in our study. The precise composition of this ribosome population is unclear, but it is likely that a large proportion of these ribosomes is translationally inactive and that translating ribosomes predominantly synthesize soluble proteins such as the Rubisco large subunit (Zoschke and Bock, 2018). Therefore, our study does not allow insight into the question whether and how the actual translation process at the membrane affects the binding of the ribosome to the translocase. Further studies using ribosomes in presence of a nascent membrane protein substrate are required to elucidate this aspect of cotranslational membrane insertion.

Our data demonstrate that the extended, negatively charged, and disordered C-terminal region of uL4c is characteristic for chloroplast ribosomes of streptophytes and absent in chlorophytes and cyanobacteria. These data indicate that the interaction between Alb3 and the C-terminus of uL4 evolved during the transition from water- to land-based plant life. Thus, this new interaction complements previously described evolutionary changes in the cotranslational pathway involving the loss of SRP RNA and the emergence of the cpSRP54/uL4 interaction during the evolutionary transition of the cyanobacterial transport to the chloroplast transport system of seed plants (Ziehe et al., 2017; Hristou et al., 2019). It can be speculated that these changes enable a higher degree of adaptability in protein transport required for the biogenesis or maintenance of the thylakoid membrane protein complexes. However, further work is required to obtain a comprehensive picture of the molecular changes in cotranslational protein transport and to understand the underlying evolutionary driving forces.

## Data Availability Statement

The original contributions presented in the study are included in the article/**Supplementary Material**, further inquiries can be directed to the corresponding author.

## Author Contributions

BA, BD, and DS contributed to conception and design of the study. BA, BD, and BJ performed the experimental work. BA and BP performed the phylogenetic analysis. BA and DS wrote the manuscript. All authors contributed to the experimental design and data analysis, and manuscript revision, read, and approved the submitted version.

## Funding

This work was supported by the Deutsche Forschungs gemeinschaft (SCHU 1163/6-2 within research unit FOR2092 and SCH 1163/7-1 within priority program 2237 MAdLand to DS, INST 213/943-1 FUGG to TGP). We acknowledge support by the Open Access Publication Funds of the Ruhr-Universität Bochum.

## Conflict of Interest

The authors declare that the research was conducted in the absence of any commercial or financial relationships that could be construed as a potential conflict of interest.

## Publisher’s Note

All claims expressed in this article are solely those of the authors and do not necessarily represent those of their affiliated organizations, or those of the publisher, the editors and the reviewers. Any product that may be evaluated in this article, or claim that may be made by its manufacturer, is not guaranteed or endorsed by the publisher.
