# Supplementary material for: Chloroplast Ribosomes Interact With the Insertase Alb3 in the Thylakoid Membrane
Source: Front Plant Sci. 2021 Dec 23;12:781857. doi: 10.3389/fpls.2021.781857 (PMC8733628; doi:10.3389/fpls.2021.781857)
Supplement: Supplementary file 1 [file data_sheet_1.zip › Supplement/SuppMaterial.docx]

Supplementary Material

# Supplementary Data

**Supplementary File 1. FASTA-file alignment of 98 uL4 sequences** (see methods and materials for details). FASTA headers all follow the syntax:
*>Species_name/Location/Taxon/ID#/Databank/SequenceLimits *underscore* Comment(optional)*
*Species_name*: Species name delimited by underscore
*Location*: CY: Cytosol CP: Plastid
*Taxon*: LP: Streptophytes GA: Chlorophytes RA: Rhodophytes CB: Cyanobacteria
*ID#*: Databank-associated ID under which the sequence is found
*Databank*: The four-letter abbreviations stand for:

- NCBI (NCBI) <https://www.ncbi.nlm.nih.gov/> [accessed: 06/07/2019]
- The Pea RNA-Seq gene atlas (PRGA) [accessed: 10/07/2019] *as of this writing defunct and embedded into the Pea Genome Project* <https://urgi.versailles.inra.fr/Species/Pisum/Pea-Genome-project>
- FernBase (FEBA) <https://www.fernbase.org/> [accessed: 08/07/2019]
- JGI-Phytozome (PHYT) <https://phytozome.jgi.doe.gov/pz/portal.html> [accessed: 08/07/2019]
- Online resource for Community Annotation of eucaryotes"OrcAE"(ORCA) <https://bioinformatics.psb.ugent.be/orcae/> [accessed: 08/07/2019]
- JGI-Phycocosm (PHYC) <https://phycocosm.jgi.doe.gov/phycocosm/home> [accessed: 08/07/2019]

*SequenceLimits*: startAA#-endAA# Relevant for cropped sequences, so signal sequences and sequencing artifacts have no influence on the Alignment.
*Comment*: excerpt of the databank annotation.

**Supplementary File 2.** **Newick-format phylogenetic tree** generated from the sequences in Supplementary file 1 (see methods and materials for details).

# Supplementary Tables

**Supplementary Table 1. Primers used for cloning of all utilized constructs**, including short descriptions of the utilized cloning strategies (see methods and materials for details). In-Fusion cloning was performed with the In-Fusion® HD EcoDry™ Cloning Plus kit (Takara Bio).

# Supplementary Figures

**Figure S1.** **Qualitative analysis of Alb3 nanodiscs. (A)** Thin layer chromatography (TLC) of lipids extracted from empty (left) or Alb3-NDs (right), compared to the asolectin stock used for ND reconstitution. Asolectin is a natural mixture of lipids, as can be seen in the different bands of the TLCs. The n-dodecyl-β-D-maltoside (β-DDM) shows up as a broad band (red arrow head), which fades after the ND preparation, showing a successful reconstitution. Adsorbent; Silica gel 60 F254 (Merck). Solvent; chloroform/methanol/H_2_O; 65 : 25 : 4 (v/v/v). Detection, UV-light after spraying the TLC plates with primuline (5 mg primuline in 100 ml acetone/water, 80:/20, v/v). S, start, F, solvent front.

**(B)** Size exclusion chromatography (SEC) of a Alb3-ND preparation (indicated in blue) and an empty ND preparation (indicated in green) with an SDS-PAGE of the peak fractions and subsequent coomassie stain. The Alb3-NDs show protein bands at 25 kDa and 45 kDa, corresponding to MSP1D1 and mAlb3 respectively. MSP1D1 eluting at around 13 ml corresponds to the size of MSP1D1-ND, while only in the presence of Alb3, part of the MSPs comigrates to the earlier elution fractions corresponding to a larger stokes-radius, where the band intensity seems to correlate with the band intensity of Alb3. Replicating this preparation without MSPs results in precipitation of Alb3 (data not shown).

**Figure S2. Full data sets of the split-ubiquitin experiments described in Figure 4 A-C.** Negative controls were performed by testing the NubG-fusions against the unrelated protein Alg5 fused to Cub-TF (Alg5: dolichyl-phosphate beta-glucosyltransferase; TF: transcription factor) and Cub-TF itself expressed from the empty bait plasmid. Cub-TF fusion constructs were controlled by using NubG-Alg5 and the unmutated form of Nub (NubI) fused to Alg5 (NubI-Alg5) as negative and positive controls respectively.

**Figure S3. Full phylogenetic tree of 98 uL4 sequences.** Unrooted maximum-likelihood tree of 98 plastidic and cytosolic uL4 sequences from chlorophytes, streptophytes, rhodophytes and cyanobacteria among 69 species (of which the 29 chlorophytes plus streptophytes are represented with both cytosolic and chloroplast sequences) inferred by using the JTT matrix-based model (1,000 bootstraps). The tree was drawn to scale with the branch lengths measured by the number of substitutions per site (black bar equals 0.1 substitutions per site). The species names are colored corresponding to Figure 5 A). The original tree is in Supplementary File 2.

**Figure S4. Full MAFFT Alignment of 98 uL4 sequence regions.** Multiple sequence alignment of 98 plastidic and cytosolic uL4 sequences from chlorophytes, streptophytes, rhodophytes and cyanobacteria among 69 species (of which the 29 chlorophytes plus streptophytes are represented with both cytosolic and chloroplast sequences). Amino acid positions are color coded by their chemical property: nonpolar hydrophobic (yellow; A,F,I,L,M,P,V,W), nonpolar hydrophilic (green; C,G,N,Q,S,T,Y), polar basic (blue; H,K,R) and polar acidic (red; D,E). The species names are colored corresponding to Figure 5 B). The original alignment is in Supplementary File 1.

**Figure S5: The uL4c C-terminus is located on the outside surface of the chloroplast ribosome and both uL4c and Alb3 possess highly disordered C-termini.** Disorder analyses of *Pisum sativum* uL4c and *Arabidopsis thaliana* Alb3 were generated, using the online disorder prediction tool PrDOS (Ishida & Kinoshita 2007). Sequence stretches that exceed the disorder probability threshold of p = 0.5 (red line) are shown as bold letters in the sequence above. **(A)** Top and bottom left: Disorder analysis of *P. sativum* uL4c. Bottom right: Structural model of the pea chloroplast ribosome (PDB: 5X8P) with the protein uL4c and the proteins uL22c, uL23c and uL29c near the exit tunnel highlighted. The location of the last C-terminal amino acids within the shown uL4c model is colored in red and indicated by the red arrow. Color coding in the sequence (bottom left) is in accordance with the structural model, showing that only amino acids 52-261 are encompassed in the model. **(B)** Disorder analysis of *A. thaliana* Alb3.
