# Supplementary material for: Chloroplast Ribosomes Interact With the Insertase Alb3 in the Thylakoid Membrane
Source: Front Plant Sci. 2021 Dec 23;12:781857. doi: 10.3389/fpls.2021.781857 (PMC8733628; doi:10.3389/fpls.2021.781857)
Supplement: Supplementary file 1 [file data_sheet_1.zip › Supplement/SuppTable 1.docx]

**Suppl. Table 1: Primers used for cloning:**

| **Construct** | **Primer** | **Cloning strategy** |
| --- | --- | --- |
| **pET29b-** |  |  |
| Alb3-C (350‑462) | for (TAAGAAGGAGATATACATATGGTGTATCTGCGTAAACTG)  rev (GTGGTGGTGGTGGTGCTCGAGCACGGTACGTTTACGTTT) | In-Fusion |
| Alb3-C Δ350‑369 | for (GGAGATATACATATGATTAGCGCGGGCCGTGCGAAACGTAGCATTGC)  rev (ACGGCCCGCGCTAATCATATGTATATCTCCTTCTTAAAGTTAAACAA) | In-Fusion; amplification of complete expression vector lacking the deleted region |
| Alb3-C Δ370‑389 | for (AACGCGAGCAAAATTCGTCAGCTGAAAGAACAGGA)  rev (TTCTTTCAGCTGACGAATTTTGCTCGCGTTTTCAT) | as above |
| Alb3-C Δ390‑409 | for (GCGGGCGAACGTTTTGTGGAACTGGTGGAAGAAAG)  rev (TTCCACCAGTTCCACAAAACGTTCGCCCGCATCAT) | as above |
| Alb3-C Δ410‑429 | for (GTGGCGAAAGATACCGAAGCGCGTGAAGGCGCGCT)  rev (GCCTTCACGCGCTTCGGTATCTTTCGCCACCGCTT) | as above |
| Alb3-C Δ430‑449 | for (GATGATGAAGAAGAACAGCGTCGTAGCAAACGTAG)  rev (TTTGCTACGACGCTGTTCTTCTTCATCATCGCTGC) | as above |
| Alb3-C Δ450‑462 | for (CTGCCGGAAGTGGGTCTCGAGCACCACCACCACCA)  rev (GTGGTGGTGCTCGAGACCCACTTCCGGCAGCGGTT) | In-Fusion |
| Alb3-C Δ451‑460 | for (TAAGAAGGAGATATACATATGGTGTATCTGCGTAAACTG)  rev (GTGGTGGTGGTGGTGCTCGAGCACGGTCTGACCCACTTCCGG) | In-Fusion |
| Alb3-C Δ397‑403 | for ([Phos]GCGGTGGCGAAAGATACCGTGGAA)  rev ([Phos]TTCCTGTTCTTTCAGCTGACGAAA) | Blunt end cloning via 5’ phosphorylation |
| Alb4-C (334‑499) | for (TAAGAAGGAGATATACATATGGTATGGCTTCAAAAATATGGTGGT)  rev (GTGGTGGTGGTGGTGCTCGAGCCTCTTCTCTGTTTCATGAGAATG) | In-Fusion |
| mAlb3ΔC (56‑349) | for NdeI (TAAGAAGGAGATATACATATGTTTAGCCTGAACGAA)  rev XhoI (GGTGGTGCTCGAGCTGCTGCGCGGTGCTC) | Classical cloning |
| mAlb4 (46‑499) | for (AAGGAGATATACATATGGCACAGCTGGGTTTTCGTCC)  rev (GGTGGTGGTGCTCGAGACGTTTTTCGGTTTCATGGCTATGC) | In-Fusion from cDNA template for codon-optimized mAlb4 |
| pAP | for NdeI (CGGCATATGAAACAAAGCACTATTGCA)  rev XhoI (TAGCTCGAGTTTCAGCCCCAGAGC) | Classical cloning |
| mAP | for NdeI (CGGCATATGCGGACACCAGAAATG)  rev XhoI (TAGCTCGAGTTTCAGCCCCAGAGC) | Classical cloning |
| mAlb3 SalI at 185‑186 | for (CAGCAGCGTTATGCGGTCGACCAGGAACGTATTCAGC)  rev (GCTGAATACGTTCCTGGTCGACCGCATAACGCTGCTG) | QuikChange mutagenesis |
| mAlb3-AP-186 | for (CAGCGTTATGCGGGCGGAGGAGGACGGACACCAGAAATG)  rev (AATACGTTCCTGGTTTCCTCCTCCTTTCAGCCCCAGAGC) | In-Fusion |
| mAlb3 SalI at 230‑231 | for (GGCGCTGAGCAACGTGGTCGACGAAGGCCTGTTTAC)  rev (GTAAACAGGCCTTCGTCGACCACGTTGCTCAGCGCC) | QuikChange mutagenesis |
| mAlb3-AP-231 | for (CTGAGCAACGTGGCGGGAGGAGGACGGACACCAGAAATG)  rev (AAACAGGCCTTCGTTTCCTCCTCCTTTCAGCCCCAGAGC) | In-Fusion |
| mAlb3-AP-304 | for (ATTATGAAACCGCCGCAGGGAGGAGGACGGACACCAGAAATG)  rev (CGCCGGATCATCGGTTCCTCCTCCTTTCAGCCCCAGAGC) | In-Fusion |
| mAlb3-AP-C | for (AAACGTAAACGTACCGTGGGAGGAGGACGGACACCAGAAATGCCT)  rev (AGGCATTTCTGGTGTCCGTCCTCCTCCCACGGTACGTTTACGTTT) | In-Fusion |
| **pETDuet** |  |  |
| eGFP | for BamHI (CGTGGATCCATGGTGAGCAAGGGCGAG)  rev EcoRI (AAAGAATTCTTGTACAGCTCGTCC) | Classical cloning |
| eGFP-uL4c (150‑282) | for SalI (TAGGTCGACAAAATCAATAGGAAGGAG)  rev NotI (CTAGCGGCCGCTTAAGCTTCCTCTGACCCTTC) | Classical cloning, ligation into the pETDuet-eGFP construct |
| eGFP-uL4c ΔC20 (150‑262) | for SalI (TAGGTCGACAAAATCAATAGGAAGGAG)  rev NotI (CTAGCGGCCGCTTAACCGTACCTCGCATTCAAGAACTCC) | as above |
| **pADSL-Nx** |  |  |
| uL4c (50‑282) | for BglII (GTTCCAGATTACGCTAGATCTGTTTCAAAACTCGGCTCC)  rev SalI (ATTACATGACTCGAGGTCGACTTAAGCTTCCTCTGACCC) | In-Fusion |
| uL4cΔC20 (50‑262) | for BglII (GTTCCAGATTACGCTAGATCTGTTTCAAAACTCGGCTCC)  rev SalI (ATGACTCGAGGTCGACTTAACCGTACCTCGCATTCAAGAACTCC) | Classical cloning |
| uL4cΔN231 (232‑282) | for BglII (GTTCCAGATTACGCTAGATCTAGGACACTGAATCTGTTTGATATTTTGAACGC)  rev SalI (AAAGTCGACTTAAGCTTCCTCTGACCCTTCCGTCTCATCTTCATCGT) | Classical cloning |
| *Synechocystis* uL4 | for BglII (GTTCCAGATTACGCTAGATCTATGGTTGATTGTATTG)  rev SalI (ATGACTCGAGGTCGACTTACTCACCGTAAACCTCC) | Classical cloning from gDNA template |
| *T. elongatus* uL4 | for BglII (GTTCCAGATTACGCTAGATCTATGGTTGCATGCGTGG)  rev SalI (ATGACTCGAGGTCGACTTAGTCACCGTACACCTCC) | as above |
| *Synechocystis* uL4+C20 | for BglII (GTTCCAGATTACGCTAGATCTATGGTTGATTGTATTG)  1. rev SalI (ATGACTCGAGGTCGACTTACGTCTCATCTTCATCGTCATCTTCTTCCTCCACCGCATACTCACCGTAAACCTCC)  2. rev SalI (AAAGTCGACTTAAGCTTCCTCTGACCCTTCCGTCTCATCTTCATCGT) | 2-step classical cloning: creation of an elongated “C+13” construct in the first step and further elongation to “C+20” in a second step by using elongating reverse primers |
| *T. elongatus* uL4+C20 | for BglII (GTTCCAGATTACGCTAGATCTATGGTTGCATGCGTGG)  1. rev SalI (ATGACTCGAGGTCGACTTACGTCTCATCTTCATCGTCATCTTCTTCCTCCACCGCATAGTCACCGTACACCTCC)  2. rev SalI (AAAGTCGACTTAAGCTTCCTCTGACCCTTCCGTCTCATCTTCATCGT) | as above |
| **pAMBV4** |  |  |
| Alb4 (46-499) | for BglII (CTATCTAGACAAAAATGGCTCAACTCGGGTTCAGA)  rev StuI (TGGAGGCCTTTCCTCTTCTCTGTTTC) | Classical cloning |
