# Supplementary figures and images for: Chloroplast Ribosomes Interact With the Insertase Alb3 in the Thylakoid Membrane
Source: Front Plant Sci. 2021 Dec 23;12:781857. doi: 10.3389/fpls.2021.781857 (PMC8733628; doi:10.3389/fpls.2021.781857)

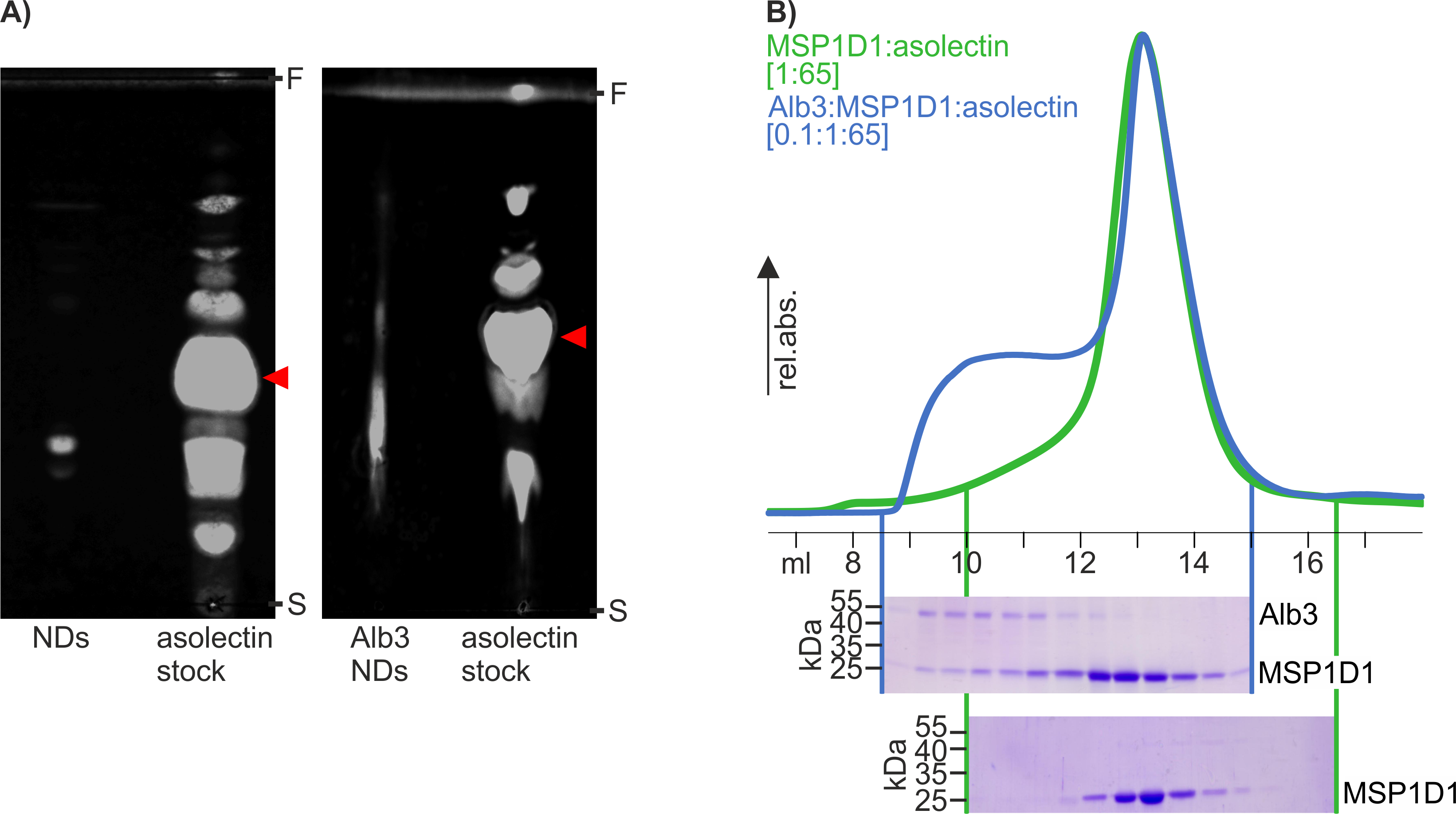

Supplement: Supplementary file 1 [file data_sheet_1.zip › Supplement/Figure S1.png]

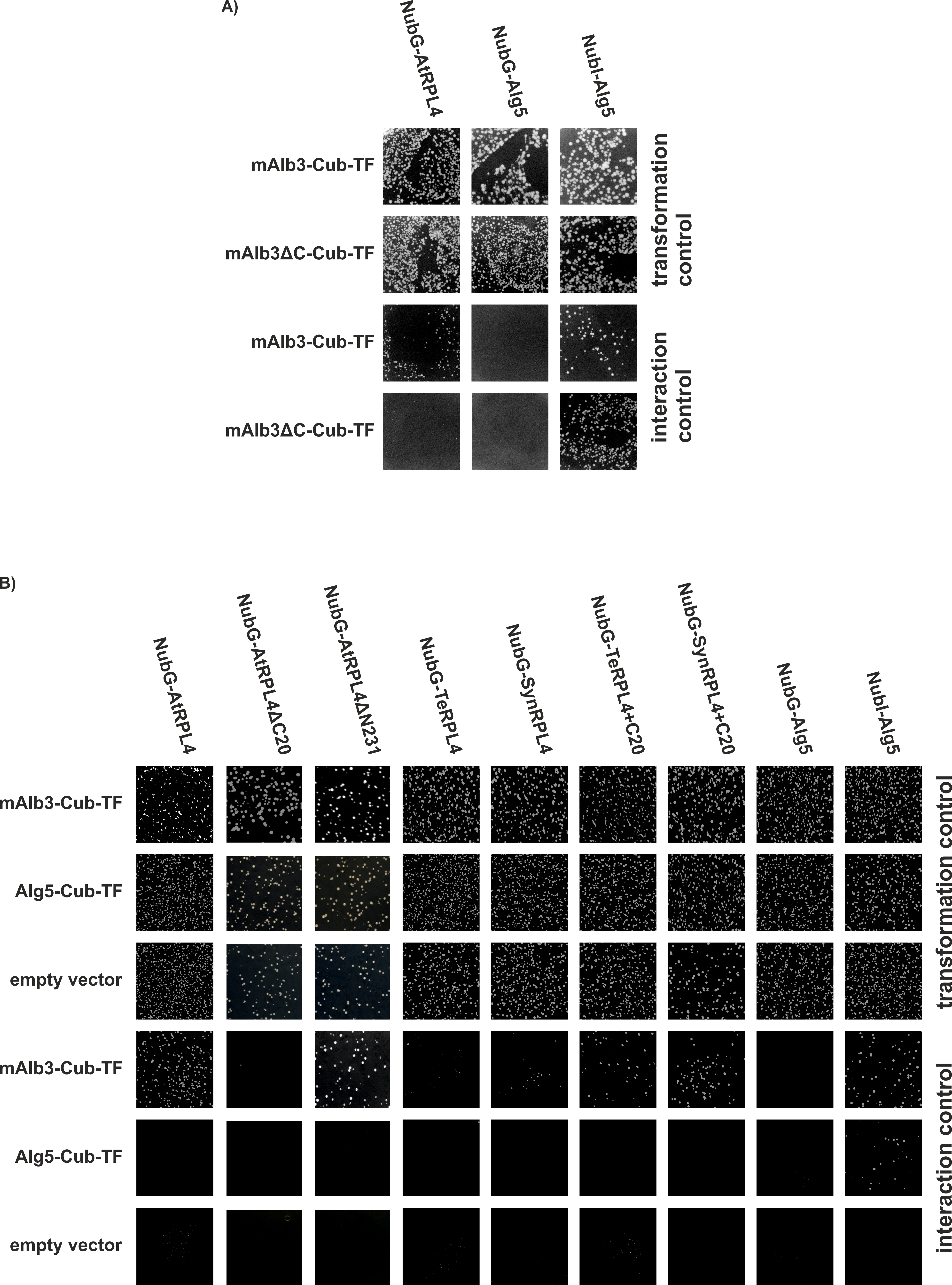

Supplement: Supplementary file 1 [file data_sheet_1.zip › Supplement/Figure S2.png]

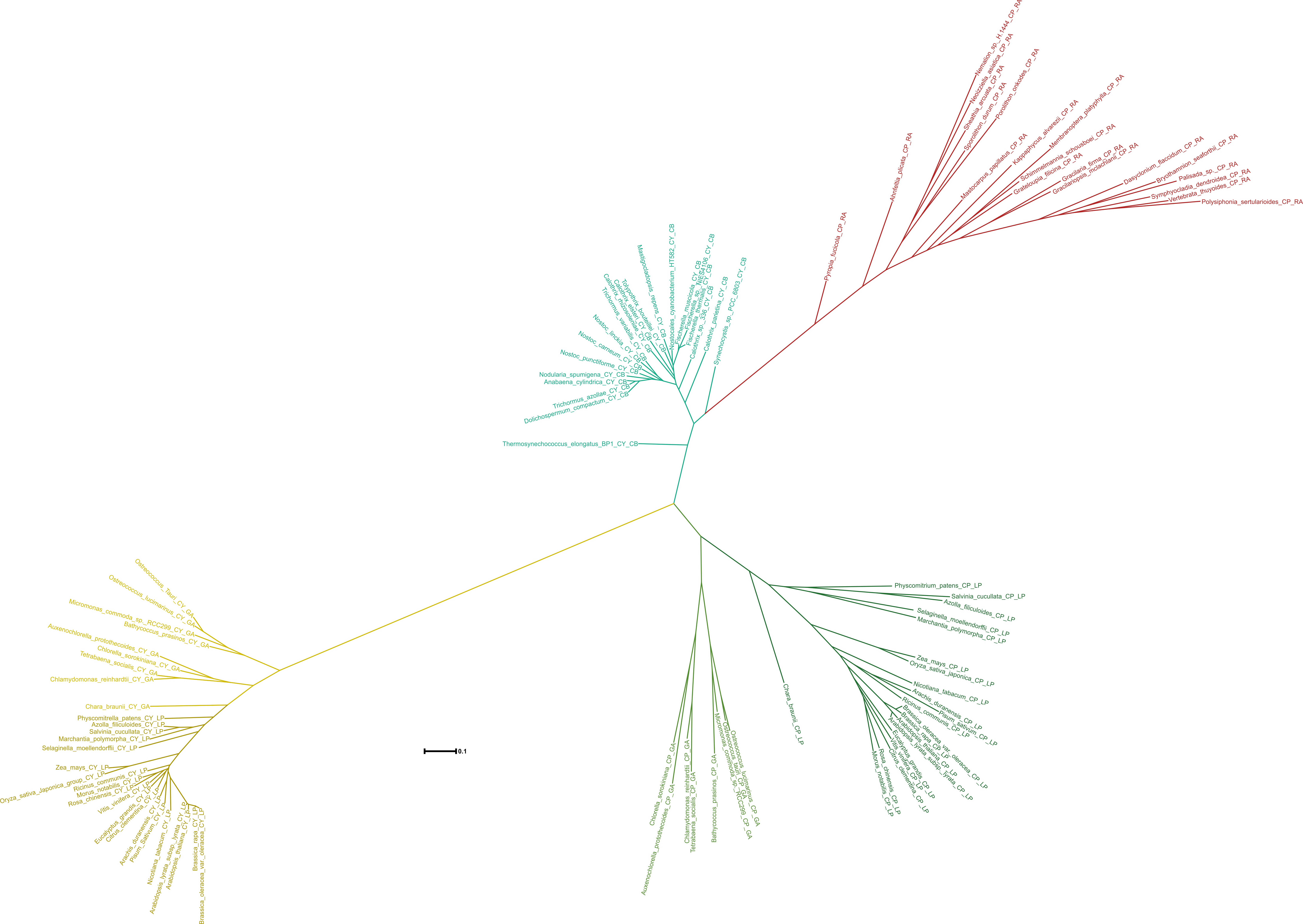

Supplement: Supplementary file 1 [file data_sheet_1.zip › Supplement/Figure S3.png]

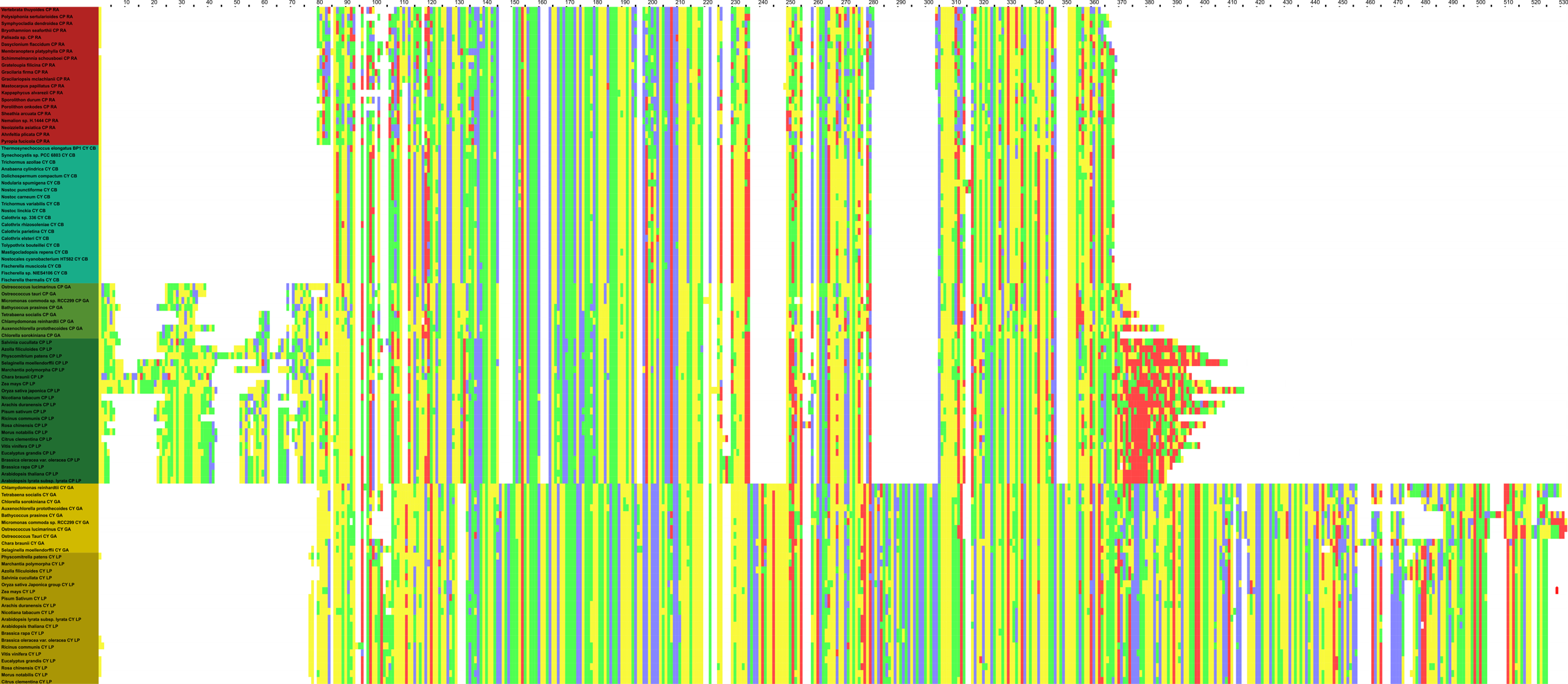

Supplement: Supplementary file 1 [file data_sheet_1.zip › Supplement/Figure S4.png]

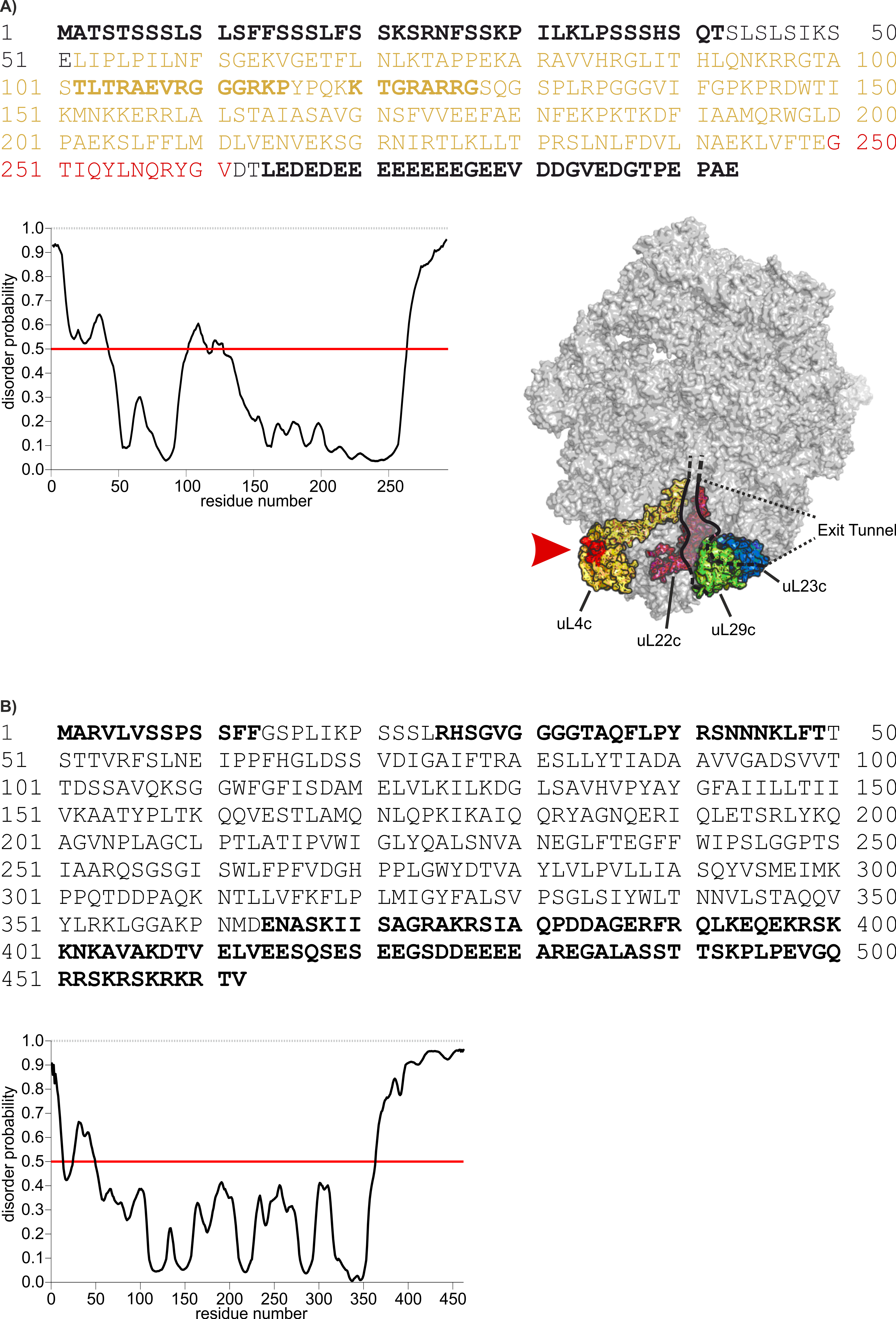

Supplement: Supplementary file 1 [file data_sheet_1.zip › Supplement/Figure S5.png]
